# Combined Barrier–Target Coverage for Directional Sensor Network

**DOI:** 10.3390/s24248093

**Published:** 2024-12-18

**Authors:** Balázs Kósa, Márk Bukovinszki, Tamás V. Michaletzky, Viktor Tihanyi

**Affiliations:** 1 TECHTRA Technology Transfer Institute, 1113 Budapest, Hungarymichaletzky.tamas@techtra.hu (T.V.M.); 2 Széchenyi István University, 9026 Győr, Hungary

**Keywords:** barrier coverage, target coverage, directional sensors

## Abstract

Over the past twenty years, camera networks have become increasingly popular. In response to various demands imposed on these networks, several coverage models have been developed in the scientific literature, such as area, trap, barrier, and target coverage. In this paper, a new type of coverage task, the Maximum Target Coverage with k-Barrier Coverage (MTCBC-k) problem, is defined. Here, the goal is to cover as many moving targets as possible from time step to time step while continuously maintaining k-barrier coverage over the region of interest (ROI). This approach is different from independently solving the two tasks and then merging the results. An Integer Linear Programming (ILP) formulation for the MTCBC-k problem is presented. Additionally, two types of camera clustering methods have been developed. This approach allows for solving smaller ILPs within clusters, and combining their solutions. Furthermore, a polynomial-time greedy algorithm has been introduced as an alternative to solve the MTCBC-k problem. An example was also provided of how the aforementioned methods can be modified to handle a more realistic scenario, where only the targets detected by the cameras are known, rather than all the targets within the ROI. The simulations were run with both dense and sparse camera placements, convincingly supporting the usefulness of the clustering and greedy methods.

## 1. Introduction

In the last two decades, camera networks, where separate cameras can operate autonomously and collaboratively, have become increasingly popular. For instance, such networks are used to monitor and manage traffic flow in urban areas, helping to reduce congestion and improve road safety. Farmers employ them to monitor crop health, detect pests, and manage irrigation systems more efficiently. They are also integral to smart city initiatives, providing data for public safety, environmental monitoring, and efficient resource management. Additionally, intruder detection is one of the most important applications of these systems, aiming to detect any trespasser attempting to penetrate the region of interest (ROI), such as national borders or critical infrastructure.

The different application fields may pose different requirements on camera networks. In certain situations, it is important to keep an eye on every point of the monitored area by one or more sensors. This is referred to as full area coverage in the related scientific literature [1]. However, achieving this may require significant resources, and in many cases, it is sufficient for the system to meet less strict conditions. For example, a network with trap coverage ensures that any moving object can travel only a limited (known) distance before it is detected by a camera. In other words, at any given moment, one can either accurately determine the exact location of a moving object or identify a coverage hole with a known diameter where it is confined [2]. Another variant of the coverage problem is k-barrier coverage. Here, the network must be deployed in such a way that any intruder or moving object crossing the ROI will inevitably pass through the field of view of *k* different cameras [3]. Possibly, the simplest version of the coverage-related problems is target coverage, where the objective is the continuous observation of static or moving objects [1]. In some cases, the targets may have different priorities [4].

In this paper, networks composed of Pan–Tilt–Zoom (PTZ) cameras are examined. In short, such cameras are equipped with motorized mechanisms that allow them to rotate horizontally and vertically and zoom in or out. The cameras are modeled as directional sensors, meaning their sensing region is represented as a sector of a circle, unlike the isotropic sensor model, which represents the sensing region as an entire circle. In practice, a PTZ camera is capable of sweeping all the sensing orientations continuously; however, the simplifying assumption is often made in scientific works that a camera can only take on a finite set of sensing directions [5]. This is also the case in this paper. The goal is to solve a combination of the barrier and target coverage tasks. This combination is inspired by the dilemma of wanting to simultaneously track the paths of already detected moving objects while not missing newly arriving ones. At national borders, a possible strategy for intruders is for one group to intentionally distract the cameras, allowing another group to slip through the temporarily unmonitored corridor unnoticed. An effective solution to the combined barrier–target coverage problem would, in addition to its other obvious advantages, also provide protection against such strategies.

Further clarifying the task to be solved, it is assumed that the barrier to be monitored is an open belt, i.e, it can be represented as an elongated rectangle [3]. The positions of the cameras are supposed to be given. The deployment can be random, as well as the result of applying a certain strategy. Camera networks often function as part of larger, heterogeneous sensor networks [6]. As a result, in addition to the cameras, the positions of moving objects are determined step-by-step with the help of other sensors. From the perspective of the task being examined, this is the ideal scenario. However, the simulations also modeled the case where cameras do not receive any additional information about the objects to be observed beyond their own detections. In the scientific literature, two types of barrier coverage are considered: strong and weak [7]. The focus here is on the strong type. In this case, regardless of how a moving object crosses the ROI, it cannot avoid detection. A brief description of weak barrier coverage is included in the Related Works section (Section 2).

When tracking a moving object, at every moment, essentially a target coverage task needs to be solved; i.e., the number of covered targets should be maximized. Meanwhile, for a predetermined *k*, k-barrier coverage must also be continuously maintained. This setup differs from solving the two tasks separately and somehow merging the results. Generally, more than one camera configuration may exist that ensures k-barrier coverage. The challenge is to find, at each time step, the optimal configuration where, on one hand, the cameras providing barrier coverage can simultaneously assist in covering targets, and on the other hand, the remaining cameras cover the remaining targets as efficiently as possible.

The contributions of this paper are as follows.

(i) A new type of coverage problem, the Maximum Target Coverage with k-Barrier Coverage (MTCBC-k) problem, is defined. The corresponding Integer Linear Programming (ILP) formulation is also presented. To the best of our knowledge, we are the first to examine this question.
(ii) A new ILP formulation for the maximum coverage problem has been provided, which is significantly different from the ones given in [8,9,10]. This formulation can be easily integrated with the part of the ILP of the MTCBC-k problem responsible for ensuring k-barrier coverage. Additionally, (a) the resulting ILP also allows for certain optimizations regarding the cameras. Examples of these optimizations include the number of cameras and the cost of their usage. (b) Furthermore, the relative importance of the targets can also be specified using the cost associated with them. (c) Finally, an upper limit can be set for the number of cameras that can be used for solving the task.
(iii) While the ILP formulation offers an optimal solution for the MTCBC-k problem, it does not scale well for large problem instances. To mitigate this difficulty, two types of camera clustering methods, horizontal and vertical, have been developed. In horizontal clustering, the clusters are formed along the possible barriers. Thus, for each cluster, the corresponding instance of the MTCBC-1 problem must be solved. The solution to the original MTCBC-k problem is given by the union of the solutions obtained from the clusters. In vertical clustering, the open belt itself is divided into sections in such a way that for each cluster, a restricted version of the original MTCBC-k problem must be solved. It is important to ensure that the chosen sectors at the cluster boundaries are directed in such a way that the complete solution provides k-barrier coverage across the entire open belt.

In addition, a polynomial-time greedy algorithm has also been introduced, consisting of two greedy subroutines. The first ensures barrier coverage. In general, it can only guarantee one-barrier coverage, but in practice, as the experiments showed, this value is often higher than one and closer to *k*. The second is responsible for target coverage and was presented in [10].

An example was also provided of how the aforementioned methods can be modified to handle a more realistic scenario, where only the targets detected by the cameras are known, rather than all the targets within the ROI. The simulations were run with both dense and sparse camera placements, convincingly supporting the usefulness of the clustering and greedy methods.

The rest of the paper is organized as follows. The related literature is presented in Section 2. In Section 3, the directional sensor model and the maximum flow problem are explained. The latter is used in the ILP formulation of the MTCBC-k problem. In Section 4, the MTC and MTCBC-k problems are defined. The corresponding ILP formulations are given in Section 5. The clustering and the greedy algorithms are explained in Section 6. In Section 7, the experiments are presented. Finally, in Section 8, the conclusions are drawn.

## 2. Related Work

One of the seminal papers that examined the question of target coverage for directional sensor networks is [8]. In this paper, the Maximum Target Coverage with Minimum Sensors (MCMS) problem was defined and shown to be NP-complete. The authors provided an ILP formulation of the task and developed centralized and greedy algorithms offering approximate solutions.

In [9], it has been proven that the Maximum Target Coverage (MTC) problem is already NP-complete in the case of directional sensors without requiring the minimality of the number of the involved sensors. This is the version of the task that is addressed in this paper. It was referred to as the directional cover set (DCS) problem in [9]. As a matter of fact, the focus of the authors of [9] was not on this question, but on an extended variant of it—the multiple directional cover sets (MDCS) problem, where they aimed to identify several possible coverings in order to be able to switch between them and thus extend the lifetime of the network.

The idea of forming clusters of sensors to more effectively solve the MCMS problem was introduced in [10]. The authors also presented a different ILP formulation of the task and offered a slightly improved centralized greedy algorithm. This is used as a subroutine in the greedy algorithm described in this paper.

In [11], a more complex form of the clustering task was examined, where the sensors within a cluster could communicate with each other, as well as sensors from different clusters. To represent message sending and receiving, the directed communication model was used, where only those neighboring sensors that lie within a specified sector could receive a message of a sensor. At first glance, it may seem that the question examined in our paper is similar to the one addressed in [11]. To ensure that each message reaches every sector, a chain needed to be formed from the communication sectors where every sector falls within a communication sector of at least one other sector. This might appear similar to the one-barrier coverage task, where a chain of the detection sectors of the sensors that runs through the observed area must be created. In addition to forming these sector chains, the maximum target coverage problem needs to be solved in both cases. In reality, however, there are several important differences between the two tasks. The most significant of these is that the communication sectors are independent of the detection sectors, thus having no impact on how the maximum target coverage problem is solved, whereas in the combined solution of barrier and target coverage tasks, a detection sector that plays a role in barrier coverage can also be crucial for target coverage. In other words, if the positions of the targets change, it has no effect on the chain formed by the communication sectors, while the chain providing barrier coverage can significantly transform. The authors of [4] present one of the earliest papers where targets were distinguished based on their importance. A higher-priority target must be monitored by multiple sensors. However, the efficiency of detection is also influenced by the distance between the target and the sensors. For example, it may be sufficient to use two sensors for a nearby target, but if the sensors are distant, three might be needed. The just described priority-based target coverage problem has gained popularity and further been examined in subsequent papers like [12]. The type of prioritization among targets enabled by the ILP formulated in our paper is different and less sophisticated compared to that introduced in [4].

Regarding the question examined, a somewhat related work to ours is [13]. Here, the goal was to cover as many moving targets as possible at each time step, and reinforcement learning was utilized to learn the optimal strategy. The effectiveness of the resulting solution was compared, among other methods, with the algorithm solving the appropriate instance of the MCMS problem at each time step.

In the context of sensor networks, the barrier coverage problem was introduced in [3]. The authors based their work on the isotropic sensor model; however, many of their results also remain valid for the directional sensor model. They distinguished weak barrier coverage from strong barrier coverage. In the latter case, regardless of how a moving object crosses the ROI, it cannot avoid detection, while in the former case, there is a given path through the ROI, and detection is only guaranteed if the object chooses a path that is congruent with this path. Two paths are congruent if one can be transformed into the other through translation and orientation.

In [14], an ILP was presented that, for given static directional sensors, determines the level of barrier coverage that the network can provide. This ILP is very similar to the part of the ILP solving the MTCBC-k problem which ensures k-barrier coverage. It is also based on the maximum flow problem over a network graph created from the coverage graph of the sectors of the sensor network. Its definition is reproduced in Section 5.3, since it is used to evaluate the efficiency of the greedy subroutine providing barrier coverage. The authors also developed polynomial-time approximating solutions to the k-barrier coverage problem. One of them, again, is similar to the greedy subroutine just mentioned. The main difference is that their algorithm is stochastic, while the greedy subroutine is deterministic.

In [15], the same question was addressed as in [14]; however, here it was assumed that the directional sensor can sweep all the directions along its circle, whereas in the previous work, the number of available directions was finite, just as it is in our work. The authors developed a polynomial algorithm to determine whether the given sensor network can provide one-barrier coverage. An energy-efficient solution was also presented that approximately minimized (1) the total and (2) the maximum rotation angles while rotating the cameras to ensure barrier coverage.

Finally, in [16], a new type of coverage problem called the target-barrier coverage problem was defined. Although the name of the problem includes both the terms barrier and target, it addresses a question entirely different from the one examined in our paper. Specifically, a target barrier is a continuous circular barrier formed around the target. It has a dbound parameter that defines the minimum distance of the constructed barrier from the target. The authors focused on how to minimize the number of members required to construct target barriers in a distributed manner while satisfying the dbound constraint and minimizing the amount of message exchange required. The target-barrier coverage problem is still an actively researched question. One of the most recent papers written on the topic is [17].

In summary, the MTCBC-k problem introduced in this paper differs from all the presented works. While it draws ideas from them, it addresses a new type of coverage problem that, to the best of our knowledge, has not been researched before.

## 3. Preliminaries

In order to work in a formal context with PTZ cameras, an appropriate model needs to be introduced. This is accomplished in Section 3.1. There and in what follows, PTZ cameras will be referred to as sensors.

To ensure k-barrier coverage, a special instance of the maximum flow problem needs to be solved. To be able to refer to the relevant concepts precisely, the definition of the problem is included in Section 3.2.

### 3.1. Directional Sensor Model

**Figure 1:**
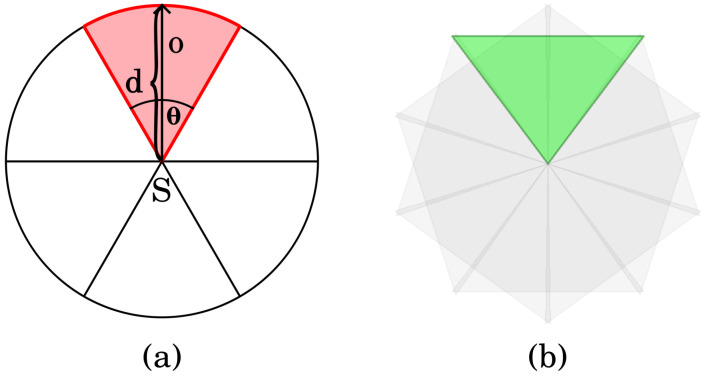
(**a**) A directional sensor with its sectors and the quadruple characterization of a sector. (**b**) A sensor and its sectors used in the experiments.

Unlike isotropic sensors, a directional sensor generally has an angle of view less than 360 degrees, so it cannot sense the entire circular area around itself. In a two-dimensional plane its sensing region can be viewed as a sector. Such a sector of sensor *S* can be characterized by a quadruple (p,o,θ,d). Here, *p* is the location of *S* given in Cartesian coordinates (x,y), while *o* and θ denote the orientation and angle of view of *S*, respectively. Finally, *d* specifies the maximum detection distance. See Figure 1a for further details.

To be able to solve the coverage and tracking problems effectively, it is assumed that a directional sensor can only take a finite set of orientations. To simplify the notation and facilitate further explanations, it is supposed that all sectors have the same angle of view and maximum detection distance, and the orientations divide the 360 degrees into equal parts. However, this is not necessary, as the algorithms to be presented work the same way even when these assumptions do not hold. For instance, during the simulations, triangles were used to represent sectors, and the individual sectors could overlap with each other (Figure 1b).

To determine when sensor *S* detects a target, the Target in Sector (TIS) test is used [1]. Informally, this test requires that the target must fall within the detection range of *S*, and it must also lie within the field of view of *S* based on its current orientation. In other words, this means that in the two-dimensional plane the position of the target must be inside or on the edge of the currently active sector of *S*. Formally:

(1) $${∥v∥}_{2}\leq d\;\mathrm{and}\;{o_{1}}^{T}v\geq {∥v∥}_{2}\cos\left(\frac{\theta }{2}\right).$$

Here, v is the vector pointing from the location of *S* to the target, ${∥v∥}_{2}$ denotes the length of v in the Euclidean space, $o_{1}$ is the unit vector with the same direction as the orientation of *S*, while *d* and θ stand for the maximum detection range and angle of view as before.

**Definition 1.**  *For a given sensor S, sector se of S and target t, se* covers *t, if Equation (1) holds for se and t. S covers t if at least one of its sectors covers t.*

### 3.2. Maximum Flow Problem

Let N=(V,E) be a network with two distinguished vertices s,t∈V being the source and the sink, respectively. Denote by $cap:E\to R^{\geq 0}$ the capacity function that determines the maximum amount of flow that can pass through an edge. A flow is a map $f:E\to R^{\geq 0}$ that satisfies two properties:

- Capacity constraints: ∀(u,v)∈E,f(u,v)≤cap(u,v);
- Conservation of flows: $\forall v\in V\setminus \left\{s,t\right\},\sum_{(u,v)\in E}f\left(u,v\right)=\sum_{(v,w)\in E}f\left(v,w\right)$. In other words, the sum of the flows entering a vertex must equal the sum of the flows exiting the same vertex, except for the source and the sink.

The *value of a flow* is defined to be the sum of the flows given on the edges originating from the source, $\sum_{(s,v)\in E}f\left(s,v\right)$. The task is to find the flow with the maximum value.

## 4. Problem Statement

### 4.1. The MTC Problem

Let $S=\{S_{1},\cdots ,S_{n}\}$ be the set of sensors. Denote by $se_{1}^{i},\cdots ,se_{r}^{i}$ the sectors belonging to $S_{i}$. Obviously, a sensor cannot look in two directions at the same time. The next definition formalizes this phenomenon.

**Definition 2.**  *With the above notations, $\mathrm{PSS}=\{se_{j_{1}}^{i_{1}},\cdots ,se_{j_{k}}^{i_{k}}\}$ is a *permissible sector selection *if there are no two sectors in PSS that belong to the same sensor.*

Let $T=\{t_{1},\cdots ,t_{m}\}$ be the set of targets. Denote $\phi_{j}^{i}$ the subset of those targets that are covered by sector $se_{j}^{i}$.

**Definition 3.**  *With the above notations, in the Maximum Target Coverage (MTC) problem, one must find the permissible sector selection coverage $\mathrm{PSS}=\{se_{j_{1}}^{i_{1}},\cdots ,se_{j_{k}}^{i_{k}}\}$ for which the cardinality of ${⋃}_{s=1}^{k}\phi_{j_{s}}^{i_{s}}$ is maximal. In other words, the permissible sector selection covering the maximum number of targets is to be found.*

### 4.2. The MTCBC-k Problem

First, the barrier coverage problem is defined. Here, an elongated rectangle, referred to as *open belt* [3], is considered where the horizontal sides are substantially longer than the vertical ones. A crossing path is defined as a path that traverses the belt from one horizontal side to the other. The idea is to place the sensors and select the active sectors in such a way that, no matter which way an object moves, it cannot avoid being detected when crossing this belt. Informally, in case of k-barrier coverage, it is guaranteed that this object is detected by *k* different sensors.

Let $S=\{S_{1},\cdots ,S_{n}\}$ be the sensors deployed on an open belt and let $\mathrm{PSS}=\{se_{j_{1}}^{i_{1}},\cdots ,se_{j_{r}}^{i_{r}}\}$ be a permissible sector selection. Let T denote the set of targets, consisting of both static and moving objects.

**Definition 4.**  *With this notation, a path l crossing the open belt is said to be* k-covered (by PSS) *if for k different sectors in PSS, $se_{j_{s}}^{i_{s}}$, $l\cap se_{j_{s}}^{i_{s}}\neq \emptyset$. The open belt is* k-barrier covered (by PSS) *if all crossing paths are k-covered (by PSS) [3].*

**Definition 5.**  *In the Maximum Target Coverage with k-Barrier Coverage (MTCBC-k) problem, one must find the permissible sector selection coverage PSS that provides k-barrier coverage on the open belt. In addition, among the permissible sector selections that guarantee k-barrier coverage, PSS must be the one covering the maximum number of targets.*

**Figure 2:**
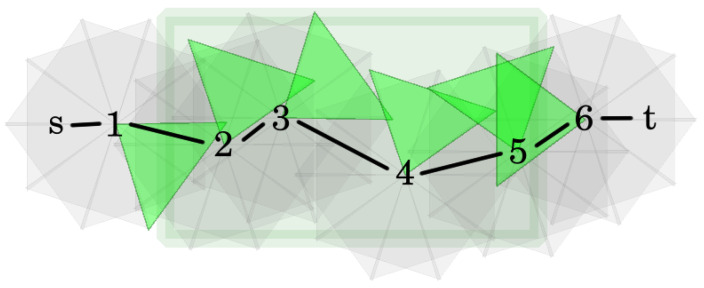
The coverage graph of a permissible sector selection.

To be able to solve the MTCBC-k problem, it is worthwhile to understand the graph-based characterization of k-barrier coverage. For a given open belt, deployed sensors, and permissible sector selection PSS, a *coverage graph* $CG_{\mathrm{PSS}}$ can be defined. The vertices of $CG_{\mathrm{PSS}}$ represent the sectors, plus two extra vertices, *s* and *t*, stand for the left and right boundaries of the belt, respectively. Two sector vertices are connected with an undirected edge if their intersection is not empty. *s* (or *t*) is connected with a sector vertex if the sector intersects the left (or the right) boundary. An example can be found in Figure 2. Exactly the same way as in [3], the following theorem can be proven.

**Theorem 1.**  *An open belt with a sensor network is k-barrier covered by permissible sector selection PSS if and only if there exist k vertex-disjoint paths from s to t in $CG_{\mathrm{PSS}}$.*

## 5. ILP Formulations

### 5.1. The MTC Problem

In this section, the ILP, $ILP_{MTC}$, which solves the MTC problem, is formulated. When tracking moving objects, $ILP_{MTC}$ should be updated and computed at each time step.

As before, let $S=\{S_{1},\cdots ,S_{n}\}$ be the sensor network deployed on the open belt. Let $T=\{t_{1},\cdots ,t_{m}\}$ be the set of targets. In addition to the actual sectors of the sensors, to each target a dummy sector is assigned. This sector covers only the given target and does not belong to any sensor. (Alternatively, it can also be said that each dummy sector belongs to a new sensor that only has this sector, or the rest of its sectors do not cover any targets.) The role of the dummy sectors will become apparent soon. If $n_{se}$ denotes the number of the real sectors, then the total number of sectors is $n_{se}+m$, where *m* is the number of targets.

The elements of the variable vector x of $ILP_{MTC}$ will represent whether a sector is selected to be active or not; hence, they can take 0 or 1 values. It is assumed that the last *m* elements of x correspond to the dummy sectors.

The cost vector, $c_{\mathrm{MTC}}$, is defined as follows:

$$c_{\mathrm{MTC}}\left[i\right]=\left\{\begin{array}{cc} 0 & \mathrm{if}\;i\leq n_{se}\\ 1 & \mathrm{otherwise}. \end{array}\right.$$

In other words, the  $i^{th}$ element of $c_{\mathrm{MTC}}$ is 0 if it represents a real sector and 1 otherwise. The aim is to minimize $c_{\mathrm{MTC}}x$, where $c_{\mathrm{MTC}}x$ is the dot product of $c_{\mathrm{MTC}}$ and x. Obviously, $c_{\mathrm{MTC}}x$ is minimized if as few dummy sectors as possible are used to cover the targets.

As constraints of $ILP_{MTC}$, two requirements need to be expressed. Firstly, it needs to be guaranteed that each solution encodes a permissible sector selection. Secondly, this permissible sector selection must cover all targets. Note that dummy sectors were introduced specifically to ensure the existence of such permissible sector selections since, clearly, a permissible sector selection containing all dummy sectors always covers all targets.

Formally, $ILP_{MTC}$ is:

$$\begin{array}{ccc} \; & \min c_{\mathrm{MTC}}x, & \;\mathrm{subject}\;\mathrm{to}\text{:} \end{array}$$

(2) $$\begin{array}{ccc} \; & \sum_{j}^{n_{se}+m}a_{ij}x_{j}\leq 1, & \;\forall i=1\cdots n, \end{array}$$

(3) $$\begin{array}{ccc} \; & \sum_{j}^{n_{se}+m}b_{kj}x_{j}\geq 1, & \;\forall k=1\cdots m. \end{array}$$

Here,

$$a_{ij}=\left\{\begin{array}{cc} 1 & \mathrm{if}\;\mathrm{the}\;j^{\mathrm{th}}\;\sec\mathrm{tor}\;\mathrm{belongs}\;\mathrm{to}\;\mathrm{the}\;i^{\mathrm{th}}\;\mathrm{sensor}\\ 0 & \mathrm{otherwise}. \end{array}\right.$$

Basically, the $a_{ij}$ elements together form the incidence matrix of the sensors and their sectors. Note that if the $j^{th}$ element of x, $x_{j}$ represents a dummy sector, then $a_{ij}$ is 0 for all sectors, i.e., for all *i*.

On the other hand,

$$b_{kj}=\left\{\begin{array}{cc} 1 & \mathrm{if}\;\mathrm{the}\;k^{\mathrm{th}}\;\mathrm{target}\;\mathrm{is}\;\mathrm{covered}\;\mathrm{by}\;\mathrm{the}\;j^{\mathrm{th}}\;\sec\mathrm{tor}\\ 0 & \mathrm{otherwise}. \end{array}\right.$$

The $b_{kj}$ elements together constitute the incidence matrix between the targets and the sectors.

It is easy to see that if Inequality (2) is satisfied, then x encodes a permissible sector selection. Meanwhile, Inequality (3) guarantees that each target is covered by at least one sector. Furthermore, the minimality condition ensures that among the permissible selections that provide a complete coverage, the ILP solutions will be those where the number of dummy sectors used is minimal, or to put it differently, the number of targets covered by real sectors is maximal. All together, this shows that a solution of $ILP_{MTC}$ encodes a solution to the MTC problem.

#### Further Considerations

Optionally, the number of usable sensors can also be limited. Simply add inequality

(4) $$\sum_{j}^{n_{se}+m}\ell_{j}x_{j}\leq r$$

to the constraints of $ILP_{MTC}$. Here,

$$\ell_{j}=1,\;\mathrm{if}\;j\leq n_{se}\;\mathrm{and}\;0\;\mathrm{otherwise}.$$

Evidently, if x is a solution to this extended version of $ILP_{MTC}$, then the encoded permissible sector selections at most *r* sectors, and consequently *r* sensors, are used.

Note also that the first $n_{se}$ elements of $c_{MTC}$ are 0, which means additional costs can be introduced for the real sectors. For example, if all these elements are set to 1 and the cost of using a dummy sector is higher than the number of the real sectors, then a solution to this modified ILP also minimizes the number of employed sensors. Alternatively, the sum of the angles of rotation required to move from one sector selection to another can be minimized as well.

Lastly, the targets can also be distinguished from each other. For example, if target *t* must be covered and there is a real sector capable of covering it, then the dummy sector corresponding to *t* should not be added to $ILP_{MTC}$. This ensures that *t* is covered by a real sector in any solution.

The targets can also be distinguished from each other based on the costs assigned to their associated dummy sectors. Suppose that the set of targets contains both static and moving targets. Then, for example, if $c_{st}=4c_{mo}$, where $c_{st}$ and $c_{mo}$ denote the costs of the dummy sectors of static and moving targets, respectively, then covering four moving targets is equally important as covering one static target.

### 5.2. The MTCBC-k Problem

In the ILP, $ILP_{MTCBC}$, which solves the MTCBC-k problem in addition to covering the maximum number of targets, k-barrier coverage must also be ensured. Recall that by Theorem 1, an open belt is k-barrier covered by a permissible sector selection PSS if and only if there are *k* vertex-disjoint paths from *s* to *t* in the coverage graph representing PSS, $CG_{\mathrm{PSS}}$. In what follows, a network graph with the appropriate capacities will be constructed in such a way that the size of the maximum flow will be equal to the maximum number of these vertex-disjoint paths (when Menger’s theorem is proven by reducing it to the Max-flow min-cut theorem, it is shown that for any undirected graph with selected vertices *s* and *t*, a network can be created where the value of the maximum flow is equal to the number of vertex-disjoint paths between *s* and *t* [18]. Here, basically, the same construction is utilized). The maximum flow problem can be formulated as a linear program, and this construction will be used in $ILP_{MTCBC}$ to guarantee k-barrier coverage.

Let B be an open belt with deployed sensor network S. Denote by T the set of targets. In the construction of the aforementioned network graph, first a coverage graph, $CG_{S}$, is created. This will be very similar to the coverage graph of a permissible sector selection. The only difference is that, in this case, not just a single permissible sector selection will be represented but all of them. Next, $CG_{S}$ will be modified in two steps. First, since capacities and flows can be defined on edges and $CG_{S}$ represents sectors as vertices, these vertices will be replaced with edges. Then, the undirected edges will be converted into directed ones.

Considering the details, as in the case of a coverage graph belonging to a permissible sector selection, the vertices of $CG_{S}$ stand for sectors of S and the left and right boundaries of the open belt. These last two vertices are denoted by *s* and *t*. The edges of *s* and *t* can be defined exactly in the same way as before. Two sector vertices are connected if the sectors belong to different sensors and their intersection is not empty.

**Figure 3:**
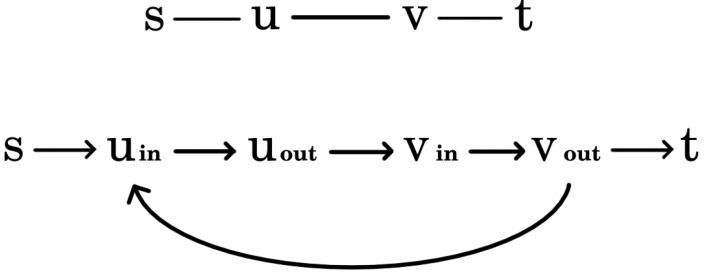
A simple example of the transformation from a coverage graph to the corresponding network graph.

Next, each sector vertex *v* of $CG_{S}$ should be substituted by a directed edge $\left(v_{in},v_{out}\right)$. Finally, the remaining undirected edges of $CG_{S}$ are changed to directed ones. Edges with *s* (or t) as an endpoint should be converted to edges originating from *s* (or ending in *t*). Additionally, each edge (u,v) between sector vertices should be replaced with two directed edges: $\left(u_{out},v_{in}\right)$ and $\left(v_{out},u_{in}\right)$. An example can be found in Figure 3. Finally, the capacity of the edges derived from sector vertices is set to 1, while the capacity of the remaining edges is defined to be infinity. Denote the resulting network as $N_{S}$.

**Definition 6.**  *With the above notations, a flow f in $N_{S}$ is* permissible *if there are no two sectors represented by the sector edges with positive flow that belong to the same sensor. Here, a sector edge is an edge $\left(u_{in},u_{out}\right)$ derived from sector vertex u in $CG_{S}$.*

**Theorem 2.**  *With the above notations, there is a permissible sector selection, PSS, in S that provides k-barrier coverage on B if and only if there exists a permissible flow of value k from s to t in $N_{S}$.*

**Proof.**  By Theorem 1, B is k-barrier covered by PSS if and only if there are *k* vertex-disjoint paths from *s* to *t* in $CG_{\mathrm{PSS}}$. From the definition of a permissible sector selection it follows that no two sectors involved in these paths belong to the same sensor. $CG_{\mathrm{PSS}}$ is a subgraph of $CG_{S}$, and hence these paths are also included in $CG_{S}$. Now, note that if $s,v_{1},\cdots ,v_{r},t$ is a path in $CG_{S}$, where $v_{1},\cdots ,v_{r}$ are sector vertices, then $s,v_{1,in},v_{1,out},\cdots ,v_{r,in},v_{r,out},t$ is a directed path in $N_{S}$ and vice versa. Thus, there exist *k* different paths from *s* to *t* in $N_{S}$ such that no two sector edges in the paths represent sectors that belong to the same sensor. The capacities of sector and non-sector edges are 1 and *∞*, respectively, and therefore on each such path a flow of value 1 can go from *s* to *t*. Since the number of these paths is *k*, this proves the existence of a permissible flow of value *k* from *s* to *t* in $N_{S}$. By reversing the above reasoning, the other direction of the statement can also be proven.    □

**Remark 1.**  *Paths in $N_{S}$ where no two sectors represented by the sector edges of these paths belong to the same sensor are called* sensor-disjoint*. Consider a flow of value k in $N_{S}$. In the proof of Theorem 2, the edges with positive flow form k sensor-disjoint paths from s to t have just been shown. Obviously, sensor-disjoint paths do not even share a common edge. This is because at least one ending vertex of each edge in $N_{S}$ represents a sector.*

Based on Theorem 2, when formulating $ILP_{MTCBC}$, the existence of a permissible flow of value *k* needs to be ensured. This in turn guarantees the existence of a permissible sector selection providing k-barrier coverage on B.

The elements of the variable x of $ILP_{MTCBC}$ stand for sector, non-sector edges of $N_{S}$, and dummy sectors as in the case of $ILP_{MTC}$. Their numbers are $n_{se},n_{e}$, and *m*, respectively, where *m* is the number of targets. It is also assumed that the elements appear in x in the aforementioned order. The first $n_{se}+n_{e}$ elements represent the flow on the sector and non-sector edges of $N_{S}$. Since the goal is to encode whether a sector is selected or not, the flow of the sector edges can be either 0 or 1, while the non-sector edges can have arbitrary non-negative flows. It follows from the Ford–Fulkerson method that if the capacities are integer or infinite values, as is the case in $N_{S}$, then there exists a maximum flow *f* such that f(u,v) is an integer for every edge (u,v) [18]. This statement guarantees that despite restricting the possible values of the “sector elements” in x to 0 or 1, a maximum flow can still be found. Finally, the last *m* elements of x represent whether a dummy sector is selected or not for covering the targets, and thus they can also have either 0 or 1 values.

The goal formulated with cost optimization is the same as in $ILP_{MTC}$: to minimize the number of dummy sectors used for covering the targets. Consequently, the cost vector, $c_{\mathrm{MTCBC}}$, is basically the same as $c_{\mathrm{MTC}}$:

$$c_{\mathrm{MTCBC}}\left[i\right]=\left\{\begin{array}{cc} 1 & \mathrm{if}\;i>n_{se}+n_{e}\\ 0 & \mathrm{otherwise}. \end{array}\right.$$

The constraints of $ILP_{MTCBC}$ represent the conservation of flows rule and the capacity constraints, and they also ensure the existence of a k-valued flow from *s* to *t* in $N_{S}$. In addition, a slight modification of Inequalities (2) and (3) should also be included in order to guarantee that a solution can only encode a permissible sector selection which covers all of the targets.

For vertex *v* of $N_{S}$, denote by In(v), Out(v) the sets of ingoing and outgoing edges of *v*, respectively. Now, $ILP_{MTCBC}$ can be defined as follows:

$$\begin{array}{ccc} \min c_{\mathrm{MTCBC}}x, & \; & \;\mathrm{subject}\;\mathrm{to}\text{:} \end{array}$$

(5) $$\begin{array}{ccc} \sum_{x_{j}\in Out\left(v\right)}x_{j}=\sum_{x_{r}\in In\left(v\right)}x_{r}, & \; & \forall v\in V.N_{S}\setminus \left\{s,t\right\}, \end{array}$$

(6) $$\begin{array}{ccc} 0\leq x_{s}\leq \infty , & \; & \forall s,\;x_{s}\;\mathrm{represents}\;a\;\mathrm{non}\text{-}\sec\mathrm{tor}\;\mathrm{edge}, \end{array}$$

(7) $$\begin{array}{cc} \sum_{p}^{n_{se}+n_{e}+m}c_{p}x_{p}=k, & \; \end{array}$$

(8) $$\begin{array}{ccc} \sum_{\ell }^{n_{se}+n_{e}+m}a_{i\ell }x_{\ell }\leq 1, & \; & \forall i=1\cdots n, \end{array}$$

(9) $$\begin{array}{ccc} \sum_{j}^{n_{se}+n_{e}+m}b_{qj}x_{j}\geq 1, & \; & \forall q=1\cdots m, \end{array}$$

where *n* and *m* are the numbers of sensors and targets, respectively, and 

$$c_{p}=\left\{\begin{array}{cc} 1 & \mathrm{if}\;\mathrm{the}\;\mathrm{edge}\;\mathrm{represented}\;\mathrm{by}\;x_{p}\;\mathrm{is}\;\mathrm{directed}\;\mathrm{from}\;s\\ 0 & \mathrm{otherwise}. \end{array}\right.$$

Thus, Equality (7) guarantees that a flow of value *k* leaves *s* in $N_{S}$, while Equality (5) and Inequality (6) ensure the arrival of this flow at *t*.

The definitions of $a_{i\ell }$ and $b_{qj}$ are essentially the same as given in Inequalities (2) and (3). The $a_{ij}$ elements together form the incidence matrix of the sensors and their sectors, while the $b_{kj}$ elements together constitute the incidence matrix between the targets and the sectors. The only difference is the presence of non-sector edges in this case. However, these have no role here. Thus, the corresponding positions can be filled with 0 s. It is easy to see now that if Inequality (8) is satisfied, then x encodes a permissible sector selection. Meanwhile, Inequality (9) guarantees that each target is covered by at least one sector.

Based on what has been discussed so far in this section, it is clear that a solution to $ILP_{MTCBC}$ indeed represents a solution to the MTCBC-k problem.

#### Further Considerations

Note that the number of sensors to be used can be limited in the same way as in the case of $ILP_{MTC}$. The same can be said about the possible cost assignment to real sectors and the opportunity to distinguish between targets.

### 5.3. Supplementary ILP Formulations

In what follows, it will be important to find a permissible sector selection PSS that provides *k*-barrier coverage on B. Moreover, among the permissible sector selections that also ensure k-barrier coverage, PSS should contain the minimal number of sectors—which entails that it also contains the minimal number of sensors. Next, it is shown how $ILP_{MTCBC}$ can be transformed into another ILP, $ILP_{bc\text{\_}min\text{\_}sect}$, whose solution encodes a permissible sector selection with the desired property.

Targets play no role in the formation of k-barrier coverage. Thus, the elements of the variable of $ILP_{bc\text{\_}min\text{\_}sect}$, x, only represent the sector and non-sector edges of $N_{S}$; i.e., the dummy sectors are not included. Inequality (9) also needs to be removed from the constraints. The rest of the constraints of $ILP_{MTCBC}$ can be preserved without any further change. In all these constraints, only 0s were present at the positions corresponding to the dummy sectors; therefore, deleting these columns does not lead to any significant changes.

The cost vector, $c_{\mathrm{bc}\text{\_}\min\text{\_}\mathrm{sect}}$, should be defined as follows:

$$c_{\mathrm{bc}\text{\_}\min\text{\_}\mathrm{sect}}\left[i\right]=\left\{\begin{array}{cc} 1 & \mathrm{if}\;x_{i}\;\mathrm{represents}\;a\;\sec\mathrm{tor}\;\mathrm{edge}\\ 0 & \mathrm{otherwise}. \end{array}\right.$$

$c_{\mathrm{bc}\text{\_}\min\text{\_}\mathrm{sect}}x$ needs to be minimized.

With this, $ILP_{bc\text{\_}min\text{\_}sect}$ is fully defined. It is easy to see that a solution of $ILP_{bc\text{\_}min\text{\_}sect}$ encodes a permissible sector selection that provides k-barrier coverage over B. Moreover, the minimization in the objective guarantees that the number of involved sectors is minimal.

#### Maximum Level of Barrier Coverage

To decide how strict the barrier coverage one wishes to ensure is, one must know the maximum level of barrier coverage that the sensor network is capable of providing. $ILP_{bc\text{\_}min\text{\_}sect}$ can easily be transformed into another ILP, $ILP_{max\text{\_}bc}$, which answers this question.

The structure of the variable of $ILP_{max\text{\_}bc}$ is the same as that in $ILP_{bc\text{\_}min\text{\_}sect}$. From the set of constraints in $ILP_{bc\text{\_}min\text{\_}sect}$, Equality (7), which guarantees the existence of a flow of value *k* leaving *s*, should be removed. The rest of the constraints can remain unchanged. As an objective, one should require that the value of the flow leaving *s* be maximal. Thus, $c_{\max\text{\_}\mathrm{bc}}$ should be defined in the same way as the coefficients of Equality (7)

$$c_{\max\text{\_}\mathrm{bc}}\left[i\right]=\left\{\begin{array}{cc} 1 & \mathrm{if}\;\mathrm{the}\;\mathrm{edge}\;\mathrm{represented}\;\mathrm{by}\;x_{i}\;\mathrm{is}\;\mathrm{directed}\;\mathrm{from}\;s\\ 0 & \mathrm{otherwise}. \end{array}\right.$$

$c_{\max\text{\_}\mathrm{bc}}x$ needs to be minimized.

Clearly, if $x^{*}$ is a solution of $ILP_{max\text{\_}bc}$, then the value of $c_{\max\text{\_}\mathrm{bc}}x^{*}$ is equal to the maximum level of barrier coverage that S can provide over B. Note that $ILP_{max\text{\_}bc}$ was already formulated in [14]. Here, it was only reiterated for the sake of completeness.

## 6. Approximations of the MTCBC-k Problem

Although the ILP formulation provides an optimal solution for the MTCBC-k problem, it does not scale well for larger instances. To handle this problem, two approaches are proposed. In the first case, clusters are formed from the sensors, which allows the ILP to be solved more efficiently due to the smaller size. The solutions obtained for the clusters are then combined to provide a solution to the original MTCBC-k problem. In the second case, a polynomial-time greedy algorithm, $Greedy_{MTCBC}$, is developed. This guarantees only one-barrier coverage instead of *k*. However, the simulations have shown that in practice, the level of the provided barrier coverage is fairly close to *k*.

### 6.1. Clustering

**Figure 4:**
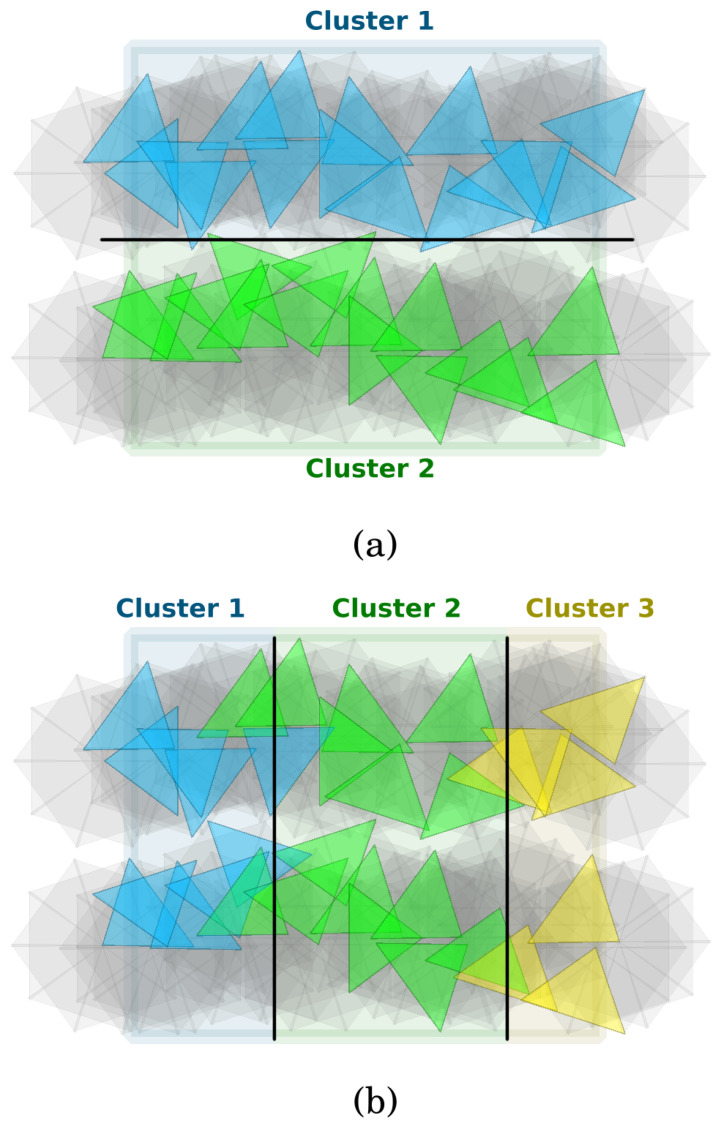
(**a**) An example of horizontal clusters. (**b**) An example of vertical clusters.

The clusters can be created both horizontally and vertically. See Figure 4a,b for examples. In the horizontal case, the main idea is to create *k* clusters in such a way that the selected sectors within each cluster provide at least one-barrier coverage on the open belt. This way, the appropriate instance of the MTCBC-1 problem needs to be solved for each cluster. The solution to the original MTCBC-k problem is given by the union of the chosen sectors.

In the vertical case, the cluster-restricted versions of the original task, which are MTCBC-k problems themselves, should be calculated. The number of clusters can be arbitrary. It is important to ensure that the chosen sectors at the cluster boundaries are directed in such a way that the complete solution offers k-barrier coverage across the entire open belt. The details will be given later.

Let B be an open belt with deployed sensor network S. In the first step, *k* sensor-disjoint paths should be found from *s* to *t* in $N_{S}$. These paths will guarantee the existence of k-barrier coverage and will form the backbone of clusters for both the horizontal and the vertical cases. However, because the paths are fixed, this will generally reduce the number of possible permissible sector selections that ensure k-barrier coverage. Hence, it is beneficial if the paths contain a minimal number of sensors, as this will allow more free sensors to be used for target coverage.

Based on Theorem 2 and Remark 1, *k* sensor-independent paths from *s* to *t* in $N_{S}$ define a permissible flow of value *k*, which in turn determines a permissible sector selection that ensures k-barrier coverage on B. Thus, the task can be reformulated as finding a permissible sector selection that ensures k-barrier coverage on B and, among such permissible sector selections, contains the minimal number of sensors. $ILP_{bc\text{\_}min\text{\_}sect}$ solves exactly this task.

#### 6.1.1. Horizontal Clusters

The pseudo-code of creating *k* horizontal clusters is given in Algorithm 1. Here, sensor_distances contains the distances of each pair of sensors. As its name suggests, get_sensor_disjoint_paths is a function that uses $ILP_{bc\text{\_}min\text{\_}sect}$ to return the sensor-disjoint paths.

In what follows, a path of $N_{S}$ is said to *contain* sensor $\hat{s}$ if *p* has a sector edge representing a sector whose sensor is $\hat{s}$. The get_sensors function returns the sensors contained by a path.

Let $S^{-}$ denote the set of those sensors that are not contained by any of the *k* sensor-disjoint paths. In the horizontal case, the sensors of a sensor-disjoint path and the sensors of $S^{-}$ that are closest to these sensors form a cluster. More precisely, for a sensor $\hat{s}$ in $S^{-}$, denote by $ns(\hat{s})$ the nearest sensor that belongs to a path; i.e., $ns(\hat{s})$ is not in $S^{-}$. For path *p*, $\hat{s}$ in $S^{-}$ is in the cluster defined by *p* if $ns(\hat{s})$ is among the sensors of *p*. get_closest_sensors returns these sensors in $S^{-}$.
**Algorithm 1** Horizontal clusters creation**Require:** $N_{S},\;\mathrm{sensor}\text{\_}\mathrm{distances},\;k$1:network_graphs←[]2:$\mathrm{paths}\leftarrow \mathrm{get}\text{\_}\mathrm{sensor}\text{\_}\mathrm{disjoint}\text{\_}\mathrm{paths}(N_{S},\;k)$3:**for** p **in** paths **do**4:    sensors←get_sensors(p)5:    $\mathrm{neighbors}\leftarrow \mathrm{get}\text{\_}\mathrm{closest}\text{\_}\mathrm{sensors}(N_{S},\;\mathrm{sensor}\text{\_}\mathrm{distances},\;\mathrm{paths},\;\mathrm{sensors})$6:    sensors←sensors∪neighbors7:    network_graphs.add(create_network_graph(sensors,s,t))8:**end for**9:**return** network_graphs

Clearly, in the network graph defined by the sensors of a cluster plus *s* and *t* (the result of create_network_graph), there is a permissible sector selection that provides one-barrier coverage on B. Thus, the MTCBC-1 problem is always solvable here. Furthermore, the union of the permissible sector selections that solves the MTCBC-1 task on the *k* clusters achieves k-barrier coverage on B, and thus it offers a solution to the MTCBC-k problem. Note, however, the number of covered targets may be less than when the original MTCBC-k task is solved, even if the MTCBC-1 problems within the clusters are solved sequentially, leaving out those targets that are covered by at least one sensor at each step.

#### 6.1.2. Vertical Clusters

The creation of vertical clusters is more complicated. The pseudo-code can be read in Algorithm 2. Since the names used are self-descriptive, the role of each function will not be explained further.

In the input parameters of the algorithm, *r* gives the number of the clusters to be created. Similar to the previous case, the output includes the network graphs associated with each cluster. As mentioned earlier, an appropriate instance of the MTCBC-k problem must be solved on each of these network graphs. However, the obtained solutions need to be connected to obtain a solution for the original MTCBC-k problem. The selected_sectors variable contains the sectors that achieve this connection. These will be active in each solution offered by the given vertical clustering.

After obtaining the *k* sensor-disjoint paths from *s* to *t*, each of them must be divided into *r* segments. In the pseudo-code, the segments variable contains these segments. It has *r* elements, and each element contains a segment from each of the *k* paths. The size of the segment of a path added to a cluster can depend on various factors. Therefore, this issue will not be discussed in detail. However, note that it is easy to write an algorithm that ensures that the number of included sensors is nearly the same for every cluster. In the simulations, this approach was chosen.

Basically, as in the previous case, for an element of segments, the sensors contained in the *k* segments and the sensors in $S^{-}$ closest to them will form a cluster (lines 8–11 in the pseudo-code). However, as has already been mentioned, the clusters must be connected to each other in this case.

**Figure 5:**
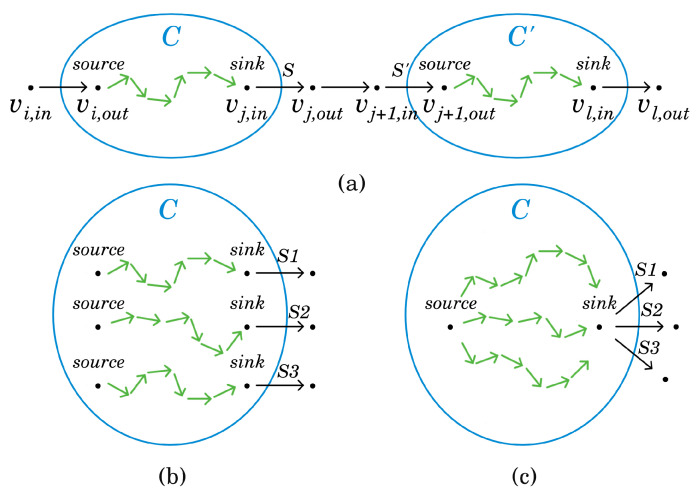
(**a**) An example for how line segments are used to form the backbone of vertical clusters. (**b**) Adding multiple line segments to a vertical cluster. (**c**) Merging the source and sink nodes.

The following explanation is technically complex, and in Figure 5 an example is provided to facilitate understanding. To grasp how such a connection can be achieved, consider one of the *k* sensor-disjoint paths, $s,v_{1,in},v_{1,out},\cdots ,v_{d,in},v_{d,out},t$. Recall that $\left(v_{i,in},v_{i,out}\right)$ is a sector edge here. Suppose that segments $p=v_{i,in},v_{i,out},\cdots ,v_{j,in},v_{j,out}$ and $p^{\prime }=v_{j+1,in},v_{j+1,out},\cdots ,v_{\ell ,in},v_{\ell ,out}$ form the basis of cluster C and $C^{\prime }$, respectively. Note that C and $C^{\prime }$ are adjacent. Furthermore, it cannot happen that the starting vertex of a sector edge belongs to one cluster while the ending vertex belongs to another cluster. Let *S* and $S^{\prime }$ be the sensors to which the sectors of $\left(v_{j,in},v_{j,out}\right)$ and $\left(v_{j+1,in},v_{j+1,out}\right)$ belong. Then, in each of the solutions of the original MTCBC-k problem based on these clusters, the sector of $\left(v_{j,in},v_{j,out}\right)$ is selected for *S*, while the sector of $\left(v_{j+1,in},v_{j+1,out}\right)$ is selected for $S^{\prime }$. In other words, these sectors are added to selected_sectors in the pseudo-code. Thus, *S* is not included in C nor $S^{\prime }$ in $C^{\prime }$. Instead, in the network graph of C, $v_{j,in}$ will be taken as the sink. Similarly, $v_{j+1,out}$ will be the source for $C^{\prime }$. In the same way, $v_{i,out}$ will be the source for C and $v_{\ell ,in}$ the sink for $C^{\prime }$. If the segment to be added starts with *s* (or ends with *t*), then *s* will be the source (or *t* will be the target). In the pseudo-code, lines 12–23 implement these operations.

For an element of segments, this procedure should be applied for each of the *k* segments (Figure 5b). Then, the resulting *k* sources (if *s* is not included) and *k* sinks (if *t* is not included) need to be merged into a single source and sink vertex, and these new vertices should be used to construct the network graph of the cluster (Figure 5c).

With this, the MTCBC-k problem to be solved in a cluster is well defined. It is also clear how the results are connected to offer a solution to the original MTCBC-k task. Again, the number of covered targets may be fewer compared to solving the original MTCBC-k task on its own. Finally, note that if horizontal clusters are created, then if a horizontal cluster is still too large, it can be further divided into vertical clusters.
**Algorithm 2** Vertical clusters creation**Require:** $N_{S},\;\mathrm{sensor}\text{\_}\mathrm{distances},\;k,\;r$1:network_graphs←initialize_array(r)2:selected_sectors←∅3:$\mathrm{paths}\leftarrow \mathrm{get}\text{\_}\mathrm{sensor}\text{\_}\mathrm{disjoint}\text{\_}\mathrm{paths}(N_{S},\;k)$4:segments←create_path_segments(paths,r)5:**for** i=0 **to** r−1 **do**6:    sensors,sources,sinks←∅,∅,∅7:    **for** j=0 **to** k−1 **do**8:        path_segment←segments[i][j]9:        sens←get_sensors(path_segment)10:      $\mathrm{neighbors}\leftarrow \mathrm{get}\text{\_}\mathrm{closest}\text{\_}\mathrm{sensors}(N_{S},\;\mathrm{sensor}\text{\_}\mathrm{distances},\;\mathrm{paths},\;\mathrm{sens})$11:      sensors←sensors∪sens∪neighbors12:      **if** i≠0 **then**13:           $\left(u_{\mathrm{in}},\;u_{\mathrm{out}}\right)\leftarrow \mathrm{first}\text{\_}\mathrm{edge}\left(\mathrm{path}\text{\_}\mathrm{segment}\right)$14:           $\mathrm{sensors}\leftarrow \mathrm{sensors}\setminus \mathrm{get}\text{\_}\mathrm{sensor}(\left(u_{\mathrm{in}},\;u_{\mathrm{out}}\right))$15:           $\mathrm{selected}\text{\_}\mathrm{sectors}\leftarrow \mathrm{selected}\text{\_}\mathrm{sectors}\cup \mathrm{get}\text{\_}\mathrm{sector}(\left(u_{\mathrm{in}},\;u_{\mathrm{out}}\right))$16:           $\mathrm{sources}\leftarrow \mathrm{sources}\cup \{u_{\mathrm{out}}\}$17:        **end if**18:        **if** i≠r−1 **then**19:           $\left(u_{\mathrm{in}},\;u_{\mathrm{out}}\right)\leftarrow \mathrm{last}\text{\_}\mathrm{edge}\left(\mathrm{path}\text{\_}\mathrm{segment}\right)$20:           $\mathrm{sensors}\leftarrow \mathrm{sensors}\setminus \mathrm{get}\text{\_}\mathrm{sensor}(\left(u_{\mathrm{in}},\;u_{\mathrm{out}}\right))$21:           $\mathrm{selected}\text{\_}\mathrm{sectors}\leftarrow \mathrm{selected}\text{\_}\mathrm{sectors}\cup \mathrm{get}\text{\_}\mathrm{sector}(\left(u_{\mathrm{in}},\;u_{\mathrm{out}}\right))$22:           $\mathrm{sinks}\leftarrow \mathrm{sinks}\cup \{u_{\mathrm{in}}\}$23:        **end if**24:    **end for**25:    source←s **if** i=0 **else** merge(sources,sensors)26:    sink←t **if** i=r−1 **else** merge(sinks,sensors)27:    network_graphs[i]←create_network_graph(sensors,source,sink)28:**end for**29:**return** network_graphs,selected_sectors

### 6.2. A Greedy Algorithm

$Greedy_{MTCBC}$ consists of two greedy methods: $Greedy_{bc\text{\_}min\text{\_}sect}$ and $Greedy_{MTC}$. The first selects as many sensor-disjoint paths as possible from *s* to *t* in $N_{S}$, but no more than *k*. It tries to choose these paths in such a way that the number of included sectors is minimal. The details of this selection will be given shortly.

Suppose now that *ℓ* such paths have been chosen. The sectors represented by the sector edges of these paths will always be active, continuously ensuring *ℓ*-barrier coverage. The remaining task from time step to time step is to select the sectors of the unused sensors that cover the maximum number of targets among those not already covered by any sector belonging to the aforementioned *ℓ* paths. This is achieved by $Greedy_{MTC}$. This algorithm was introduced in [10]. Its pseudo-code is given in Algorithm 3.
**Algorithm 3** 
$Greedy_{MTC}$
**Require:** sensor_disjoint_paths,sensors,targets1:selected_sectors←[]2:**for** p **in** sensor_disjoint_paths **do**3:    targets←targets∖get_covered_targets(p)4:**end for**5:**while** sensors≠∅∧targets≠∅ **do**6:    best←(None,−1)7:    **for** sens **in** sensors **do**8:        $n_{t,\mathrm{sens}}\leftarrow \mathrm{length}\left(\mathrm{get}\text{\_}\mathrm{covered}\text{\_}\mathrm{targets}\left(\mathrm{sens}\right)\right)$9:        **for** sect **in** sens.sectors **do**10:           $n_{t,\mathrm{sect}}\leftarrow \mathrm{length}\left(\mathrm{get}\text{\_}\mathrm{covered}\text{\_}\mathrm{targets}\left(\mathrm{sect}\right)\right)$11:           **if** $n_{t,\mathrm{sect}}/n_{t,\mathrm{sens}}>\mathrm{best}\left[1\right]$ **then**12:               $\mathrm{best}\leftarrow (\mathrm{sect},\;n_{t,\mathrm{sect}}/n_{t,\mathrm{sens}})$13:           **end if**14:        **end for**15:    **end for**16:    best_sector←best[0]17:    selected_sectors.add(best_sector)18:    sectors←sectors∖best_sector.sensor19:    targets←targets∖get_covered_targets(best_sector)20:**end while**21:**return** selected_sectors

In this pseudo-code, the sensors variable is the set of those sensors that are not contained by any of the paths of the sensor-disjoint paths from *s* to *t*. On the other hand, targets is the set of targets whose positions are known in a given time step.

First, those targets are removed that are covered by a sensor contained by a path of the sensor-disjoint paths.

Next, for a sector sect and its sensor sens, denote by $n_{t,sect}$ the number of those uncovered targets that sect can cover. Similarly, denote by $n_{t,sens}$ the number of those uncovered targets that are covered by at least one sector of sens. Consider $n_{t,sect}/n_{t,sens}$, and select that sector for which this ratio is the maximal. If there are multiple such sectors, choose one at random. The targets covered by the selected sector are considered covered in the further steps. For the remaining sensors and uncovered targets continue this procedure until there are no sensors left with inactive sectors or there are no uncovered targets.

Returning to the question of how the sensor-disjoint paths from *s* to *t* in $N_{S}$ are selected, the pseudo-code of $Greedy_{bc\text{\_}min\text{\_}sect}$ is provided in Algorithm 4. In each step, the shortest path, *p*, from *s* to *t* in $N_{S}$ should be found, ensuring that no two sector edges have the same sensors. This can be achieved by a modified Breadth-First Search (BFS), whose pseudo-code is given in Algorithm 5 and will be explained shortly. Since the result will contain sensor-disjoint paths, all edges can be deleted from $N_{S}$ that have an ending vertex $v_{in}$ or $v_{out}$ such that the represented sector belongs to a sensor contained by *p*. In the next step, this modified $N_{S}$ is considered. The algorithm stops when *k* paths have been found or there are no more paths from *s* to *t* with the desired property.
**Algorithm 4** 
$Greedy_{bc\text{\_}min\text{\_}sect}$
**Require:** $N_{S},\;k$1:selected_paths←[]2:$\mathrm{path}\leftarrow \mathrm{modified}\text{\_}\mathrm{bfs}(N_{S})$3:**while** path≠None∧length(selected_paths)<k **do**4:    selected_paths.add(path)5:    $N_{S}\leftarrow \mathrm{delete}\text{\_}\mathrm{sensors}\left(N_{S},\;\mathrm{get}\text{\_}\mathrm{sensors}\left(\mathrm{path}\right)\right)$6:    $\mathrm{path}\leftarrow \mathrm{modified}\text{\_}\mathrm{bfs}(N_{S})$7:**end while**8:**return** selected_paths

In the pseudo-code of the modified BFS (Algorithm 5), Q is a queue that stores pairs. The first element of such a pair is a vertex; denote it by *u*. The second is the set of sensors belonging to the path from *s* to *u*. child_parent is a dictionary where child_parent[v]=u means that *u* is the parent of *v* in the tree obtained during the BFS traversal. When *t* is reached first during the BFS, get_path obtains the corresponding path from *s* to *t* by means of child_parent. v∉child_parent is true if *v* is not a key in child_parent. In this context, it means that *v* has not been processed yet.

Suppose that *u* is the current vertex whose children are considered one after the other. In the pseudo-code, the curr variable serves this role. The key idea of the modified BFS is not to include a child *v* if its sensor is in the set of sensors belonging to the path from *s* to *u*. Note that if (u,v) is a sector edge, this cannot happen.

**Figure 6:**
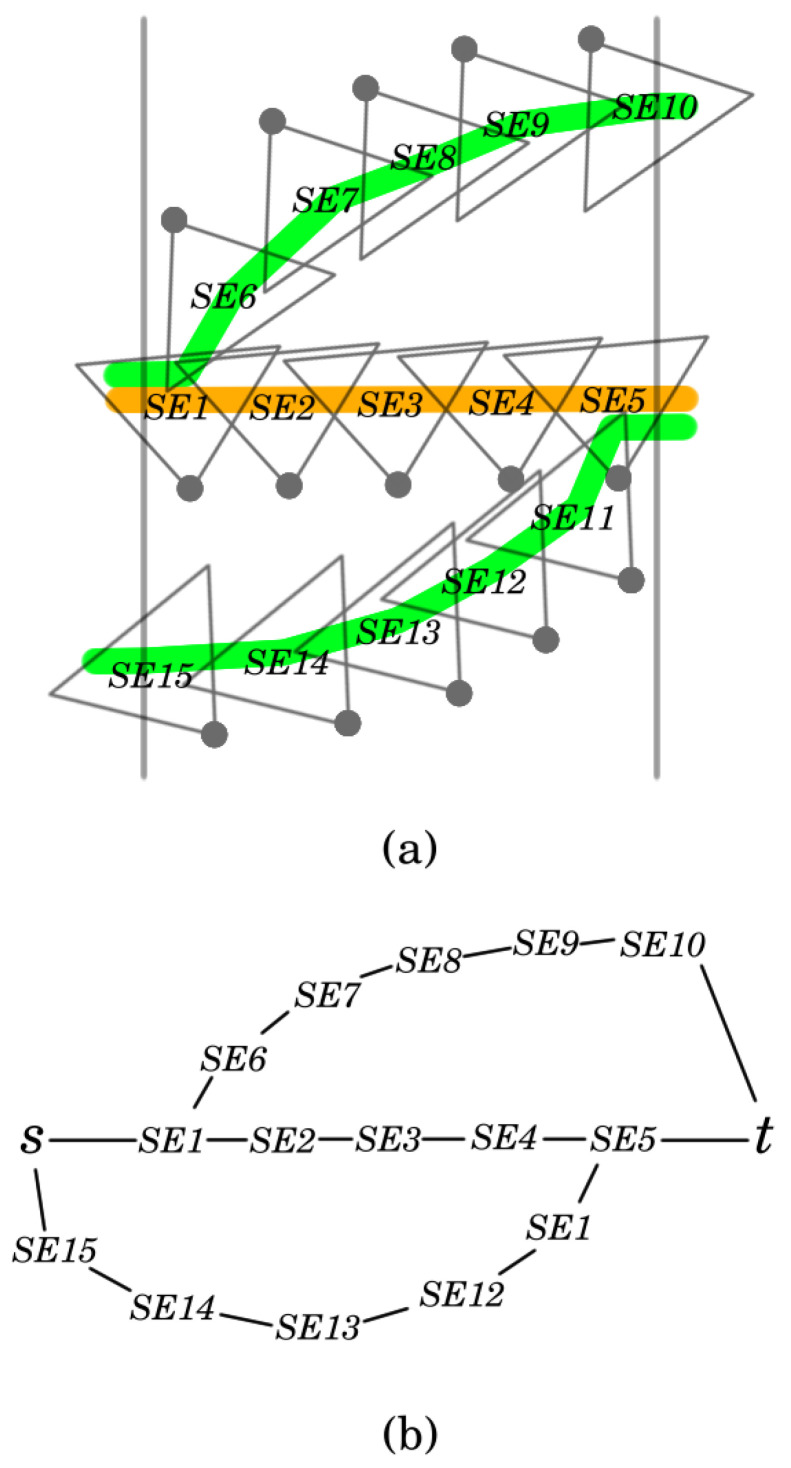
An example when $Greedy_{bc\text{\_}min\text{\_}sect}$ does not return an optimal result. (**a**) Sector. (**b**) Network graph.

$Greedy_{bc\text{\_}min\text{\_}sect}$ does not necessarily return an optimal solution. In Figure 6, one can see an example where it finds a single sensor-disjoint path from the source to the sink, while the maximum number of such paths is 2. The first experiment will provide insight into the practical effectiveness of $Greedy_{bc\text{\_}min\text{\_}sect}$.
**Algorithm 5** Modified BFS for the greedy algorithm**Require:** $N_{S}$1:Q←∅2:Q.add((s,∅))3:child_parent←{}4:**while** Qisnotempty **do**5:    curr,sensors←Q.dequeue()6:    **for all** childvofcurr **do**7:        **if** v∉child_parent **then**8:           **if** (curr,v)isasectoredge **then**9:               child_parent[v]←curr10:               Q.add(v,sensors)11:         **else**12:               **if** get_sensor((curr,v))∉sensors **then**13:                   child_parent[v]←curr14:                   **if** v==t **then**15:                       path←get_path(child_parent)16:                       **return** path17:                   **else**18:                       v_sensors←sensors∪sensor((curr,v))19:                       Q.add(v,v_sensors)20:                   **end if**21:               **end if**22:           **end if**23:        **end if**24:    **end for**25:**end while**26:**return** None

#### Runtime Analysis

Assume now that $Greedy_{bc\text{\_}min\text{\_}sect}$ finds *ℓ* sensor-disjoint paths from *s* to *t* in $N_{S}$. Since membership in a set can be determined in O(1) time, the running time of the algorithm is $O(\ell |E.N_{S}|)$, where $|E.N_{S}|$ denotes the number of edges in $N_{S}$. If *n* is the number of sensors and *q* is the number of sectors belonging to a sensor, then $|E.N_{S}{|\leq \left(nq\right)}^{2}$. In other words, the runtime of $Greedy_{bc\text{\_}min\text{\_}sect}$ is $O(n^{2}q^{2})$. It should be run only once as the first step of $Greedy_{MTCBC}$.

For the analysis of the runtime of $Greedy_{MTC}$ at a given time step, recall that the number of targets is denoted by *m*. In the first step, the covered targets for each sector of the sensor-disjoint paths from *s* to *t* should be identified. For a given sector and target, the TIS test (Equation (1)) can be accomplished in O(1) time. Thus, this step requires O(mnq) time. Note that if the objects move slower, then, relying on the knowledge of which sector covered which targets at time step t−1, at time step *t*, the number of candidate sectors covering a target can be reduced to a constant. Hence, in this case, the running time is only O(m).

After the removal of the covered targets, for each sector that is not included by any of the sensor-disjoint paths from *s* to *t*, the set of covered targets should be calculated. Again, this can be accomplished in O(mnq) or O(m) time. Based on this, the values of $n_{t,sect}$, $n_{t,sens}$ and the maximum of the ratios, $n_{t,sect}/n_{t,sens}$, can be calculated in O(nq) time. Suppose that the sector to be activated covers *r* targets. After excluding these targets, the new values of $n_{t,sect}$ can be obtained in O(rnq) time. Or, if the number of sectors that can cover a target is constant, the required time to accomplish this step is only O(r). Altogether, at a given time step, the running time of $Greedy_{MTC}$ is $O(mn^{2}q)$ or $O(n^{2}q+nm)$.

## 7. Experiments

### 7.1. Effectiveness of $Greedy_{bc\text{\_}min\text{\_}sect}$

In Figure 6, one can see an example where $Greedy_{bc\text{\_}min\text{\_}sect}$ does not return an optimal solution. To gain an idea of how effective $Greedy_{bc\text{\_}min\text{\_}sect}$ actually is, the maximum number of sensor-disjoint paths from the source to the sink was compared in 102 cases with the number of such paths found by the algorithm. More precisely, since the input of the method specifies the number of sensor-disjoint paths to be found from the source to the sink, it was assumed that this number is always equal to the maximum number of such paths. This number was obtained by applying $ILP_{max\text{\_}bc}$. Thirty sensors were placed randomly on the rectangle-shaped ROI according to uniform distribution and only those cases were taken into account where the sensors provided at least one-barrier coverage.

The results can be found in Table 1. The row and column headers show the numbers of sensor-disjoint paths returned by $ILP_{max\text{\_}bc}$ and $Greedy_{max\text{\_}bc}$, respectively. The $a_{ij}$ field of the table gives the number of cases where the results returned by $ILP_{max\text{\_}bc}$ and $Greedy_{max\text{\_}bc}$ were *i* and *j*. The data show that in 29.4% of cases, the greedy algorithm provided the optimal solution; in 60.8% of cases, it found 1 fewer sensor-disjoint paths; and in 9.8% of the cases, it returned 2 fewer. Notably, the difference between the optimal solution and that of $Greedy_{max\text{\_}bc}$ was never greater than 2.

### 7.2. Performance of the Algorithms Solving the MTCBC-k Problem

In the next experiment, the performance of different $ILP_{MTCBC}$ variants—without clusters, horizontal clustering, and vertical clustering—was compared with each other, as well as with $Greedy_{MTCBC}$ and two baseline methods. The first baseline algorithm, abbreviated as BC, ensured k-barrier coverage but did not perform maximum target coverage. In detail, in the first step, the sectors encoded by the result of $ILP_{bc\text{\_}min\text{\_}sect}$ were set to active. For those sensors that were not selected this way, a random sector was chosen. This configuration was never changed afterwards. On the other hand, the second baseline method, abbreviated as MTC, performed maximum target coverage without ensuring barrier coverage.

During the simulations, two types of scenarios were examined. In the first, the positions of all targets were known at each step, while in the second, the algorithms could only use the positions of targets covered by a sector at that moment. In what follows, the two scenarios will be referred to as *omniscient* and *only-camera*. For all methods, the implementation varied slightly between the two scenarios.

In the only-camera scenario, all methods were modified to ensure that each sensor scanned its surroundings whenever possible. Specifically, for each sensor whose active sector was not determined by the algorithm, the sector consecutive to the currently active sector was selected to be active in the next time step. In addition, for the ILP variants, k-barrier coverage was ensured with the minimal number of sensors in order to increase the number of those sensors that could scan their surroundings.

In the omniscient scenario, only the ILP variants were modified in such a way that the permissible sector selection with the least number of changes was preferred.

The performance was analyzed based on two different criteria. In both cases, for each target, only those time steps were taken into account when it was in the ROI and was within the detection distance of at least one sensor. In the first case, tracking efficiency was measured. For each target, the number of time steps during which it was covered was divided by the total number of time steps considered for that target. Then, the average of these ratios was taken. The resulting measure is referred to as the *average tracking ratio* in the sequel.

The second performance indicator, *average coverage ratio*, characterizes the efficiency of coverage. Here, in each time step, the number of covered targets was divided by the number of all targets to be considered in that time step. After this, the average of these ratios was taken.

In the simulations, one pixel represented one meter. There were 100 targets at each time step on the field. They moved two meters per time step randomly, but with a downward tendency. The simulations ran until 1000 targets completely left the belt. Once a group left the field, a new random group was generated. The detection distance of the sensors was 100 m. Three sensor setups were tested, referred to as DENSE, SPARSE, and NARROW. The parameters of these setups can be found in Table 2. In the DENSE setup, the sensors were placed in a 3×10 grid. Then, each sensor position was independently shifted by a vector drawn from the $N(0,Diag_{\sigma })$ normal distribution. Here, $Diag_{\sigma }$ denotes the 2×2 diagonal matrix where both diagonal elements are σ. The resulting sensor network was capable of providing three-barrier coverage. The placement of the sensors was performed in a similar manner for the other two setups as well. Note that the area of the belt was the same for all setups. In the case of vertical clustering, two clusters were created. Clearly, for the MTCBC-1 task, the single horizontal cluster contains all sensors, and thus this version of the algorithm is not different from the one without clusters.

The average runtime of the algorithms is given in Table 3. For each method, what percentage of the runtime of the optimal algorithm its own runtime represents is indicated. Optimal refers to that algorithm when $ILP_{MTCBC}$ is solved in each time step without clustering. The rest of the names are self-descriptive.

The data clearly show that combining the barrier and maximum target coverage problems comes at a cost, with the increased ILP causing a slowdown of 20 times or more. If the computations can be run in parallel on clusters, the runtime can be reduced to approximately half of the optimal time with two clusters. In the case of vertical clustering, which sector will be active for some sensors is always fixed, so the task to be solved was smaller than in the case of horizontal clustering (the number of clusters was the same for both cases). As a result, the runtime of vertical clustering was slightly better. Finally, it is noteworthy that the greedy algorithm runs approximately 50 times faster than the optimal one.

The values of the average coverage and tracking ratios can be found in Table 4 and Table A1. As it turned out, these values were very close to each other in almost all cases, and therefore the table with the average tracking values was placed in Appendix A. In these tables, DENSE(2) refers to that case when the MTCBC-2 problem was to be solved with the DENSE setup. The other names can be interpreted in the same way.

As expected, each method performed worse in all setups when two-barrier coverage was required, compared to when single-barrier coverage needed to be maintained. The only exception was the MTC algorithm, which is agnostic to the number of barriers by definition. This is because in the latter cases, fewer sensors were needed to form the barriers, and consequently more sensors were available to cover the targets. For a similar reason, in the SPARSE setups, where the number of cameras was lower, the performance of all the algorithms was also worse.

In terms of details, it is noteworthy that the performance of the horizontal clustering, except for the omniscient SPARSE(2) case, was at most 1–2% lower than that of the optimal method. The calculations ran in parallel on the individual clusters, so it could happen that a target was covered by sensors from both clusters, leaving other targets uncovered, which the optimal method was able to cover. If the cameras are densely placed, this is less of a problem, because even if a sensor unnecessarily covers a target, the sensors nearby can still be used to cover the other targets. However, if the number of cameras is lower, the unnecessary multiple coverages result in a noticeable performance degradation, as evidenced by the 10% performance difference in favor of the optimal method in the SPARSE(2) setup in the omniscient scenario. In the only-camera scenario, however, multiple coverages, even if they occurred, did not cause significant performance degradation. This indicates that in the SPARSE(2) setup, the optimal method was aware of roughly the same target positions as the horizontal clustering.

It is also notable that horizontal clustering outperformed the vertical one in all setups. Multiple coverages can also occur in the case of vertical clustering, so this does not necessarily cause a difference in the performance of the two types of clustering. Thus, the difference in efficiency shows that fixing the sectors, which join the results obtained on the vertical clusters, and therefore losing flexibility, comes at a price. This becomes even more apparent in the case of the SPARSE(2) setup, where the number of possible different barrier coverage configurations was the lowest. On the other hand, there are certainly scenarios in the real world where vertical clustering performs as well as or even better than horizontal clustering. Additionally, the number of vertical clusters can be arbitrary, while the number of horizontal clusters can be at most *k*, when k-barrier coverage should be maintained.

Interestingly, the performance of the greedy method was quite similar to that of vertical clustering, while its runtime was 25 times faster. Additionally, its performance was less influenced by how well the positions of the targets were known. On average, the coverage performance of the optimal method was 0.90 in the omniscient scenario and 0.76 in the only-camera scenario, which is a 15% drop. On the other hand, the performance of the greedy method dropped only by 9% from 0.75 to 0.69.

As expected, the MTC algorithm achieved the best coverage ratios in all cases, thanks to the fact that all cameras could be used to cover the targets. Averaging across the setups, it could reach coverage ratios of 0.93 in the omniscient scenario and 0.82 in the only-camera scenario. Not surprisingly, the BC method performed the worst, achieving only a 0.43 coverage ratio on average.

Based on these results, a new metric, called *relative coverage ratio*, can be introduced that illustrates the performance of each algorithm compared to the best and worst methods. In this way, it also helps to examine the relative effectiveness of the algorithms compared to each other.

Formally, if $ACR_{BC}$ and $ACR_{MTC}$ denote the average coverage ratios for the BC and MTC algorithms, the relative coverage ratio rCR for any method is calculated as

(10) $$rCR=\frac{ACR-ACR_{BC}}{ACR_{MTC}-ACR_{BC}}\cdot 100\%,$$

where ACR denotes the average coverage ratio of the method.

The relative coverage values of the algorithms averaged on all setups can be found in Table 5. Note that the *relative tracking ratio* metric can be introduced in a similar way. The corresponding values are given in Table A2. The data do not offer new insights compared to what has already been stated, but they illustratively demonstrate the relative performance of the methods.

## 8. Conclusions and Future Work

In summary, this paper introduced the Maximum Target Coverage with k-Barrier Coverage (MTCBC-k) problem, aiming to cover as many moving targets as possible in each time step while maintaining continuous k-barrier coverage over the ROI. This integrated approach contrasts with solving the two tasks separately and then merging the outcomes. Multiple camera configurations can typically ensure k-barrier coverage, presenting the challenge of finding the optimal configuration at each time step. This configuration allows cameras providing barrier coverage to also assist in target coverage, while other cameras efficiently cover the remaining targets. An ILP formulation of the MTCBC-k problem was presented, along with two types of camera clustering methods, horizontal and vertical, enabling the solution of smaller ILPs within clusters and combining their solutions. Additionally, a polynomial-time greedy algorithm was proposed as an alternative method to address the MTCBC-k problem.

The experiments clearly showed that combining the barrier and maximum target coverage problems comes at a cost. The increase in the size of the ILP to be solved caused a slowdown of 20 times or more compared to when barrier coverage or maximum target coverage was separately addressed. However, if the computations are run in parallel on clusters, the runtime can be reduced to approximately 1/r of the optimal time with *r* clusters.

It also became clear, as expected, that the stronger the barrier coverage required, the more cameras need to be used to ensure it, which leads to a decrease in the target coverage ratio.

During the simulations, two types of scenarios were considered. In the first, the positions of all targets were known at each step, while in the second, the algorithms could only use the positions of targets covered by a camera at that moment. In the second case, all methods were modified in such a way that each sensor scanned its surroundings whenever it was possible. In this way, an example was provided of how the developed algorithms can be modified to handle a more realistic scenario.

The optimal algorithm, which solved the ILP corresponding to the MTCBC-k problem by considering all the data, consistently outperformed the clustering and greedy methods. However, in the case of horizontal clustering, the performance difference was less significant in most of the setups.

Since it typically involves fewer constraints, horizontal clustering generally performs better than vertical clustering. However, in the former case, the number of possible clusters is limited to at most *k* if the goal is to ensure k-barrier coverage, while in the latter case, the number of clusters can be arbitrary.

The performance of the greedy algorithm was noticeably worse than the optimal solution but came close to that of vertical clustering. On the other hand, it runs 50 times faster than the optimal method. Additionally, its performance was less influenced by how well the positions of the targets were known.

In future work, the challenges and solutions related to the MTCBC-k problem in practical applications can be further explored, such as how camera networks should be deployed in real-world environments to optimize the real-time performance of the algorithms. Another interesting aspect could be how hybrid sensor networks, which include mobile sensors, can be used to ensure k-barrier coverage in cases where cameras alone would not be able to provide it while also helping to efficiently cover the targets, all with the goal of minimizing the movement distance of the mobile sensors. Additionally, combining the MTCBC-k problem with other related problems, such as path planning and target tracking with sensor data fusion, could be considered.

## Figures and Tables

**Table 1:** The result of comparing the outputs of $ILP_{max\text{\_}bc}$ and $Greedy_{max\text{\_}bc}$ in 102 random scenarios.

Greedy	1	2	3	4
ILP				
1	2	0	0	0
2	3	8	0	0
3	0	14	14	0
4	0	6	28	6
5	0	0	4	17

**Table 2:** Parameters of the different experimental setups.

Setup	Tasks	Size of the Belt	Number of Sensors	Max Possible Barrier Coverage
DENSE	MTCBC-1, -2	750×550	30	3
SPARSE	MTCBC-1, -2	750×550	20	2
NARROW	MTCBC-1	1650×250	30	2

**Table 3:** Runtime of the algorithms.

	Omniscient	Only-Camera
Optimal	100%	100%
Horizontal	63%	54%
Vertical	52%	49%
Greedy	2.1%	1.7%
BC	2.5%	2.1%
MTC	5.6%	3.3%

**Table 4:** Results for average tracking ratios.

Average Coverage Ratio							
Scenario	Setup	BC	MTC	Optimal	Horizontal	Vertical	Greedy
	DENSE(1)	0.4774	0.9598	0.9587	-	0.9309	0.8945
	SPARSE(1)	0.3498	0.8816	0.8606	-	0.7029	0.6626
omniscient	NARROW(1)	0.5503	0.9617	0.9605	-	0.9285	0.9004
	DENSE(2)	0.45	0.96	0.9564	0.9382	0.8751	0.8485
	SPARSE(2)	0.3184	0.8817	0.7841	0.6818	0.4978	0.4454
	DENSE(1)	0.4648	0.8898	0.8732	-	0.8244	0.8231
	SPARSE(1)	0.3476	0.7348	0.6672	-	0.5887	0.5775
only-camera	NARROW(1)	0.5503	0.9177	0.9022	-	0.8442	0.8537
	DENSE(2)	0.45	0.8903	0.8483	0.8297	0.7544	0.7515
	SPARSE(2)	0.3216	0.7351	0.5401	0.534	0.4339	0.4192

**Table 5:** Relative coverage ratio values for the omniscient and only-camera scenarios, calculated as Equation (10), averaged on all five setups. (Values of the horizontal clustering with 1 barrier were considered to be the same as those of the optimal method.)

Detection Mode	BC	MTC	Optimal	Horizontal	Vertical	Greedy
omniscient	0.0%	100.0%	95.50%	86.65%	73.51%	66.21%
only-camera	0.0%	100.0%	83.55%	77.79%	64.64%	63.68%

## Data Availability

The algorithms described in the article are parts of a larger program; for business reasons, we have not made the entire system open-source.

## Appendix A

**Table A1:** Results for average tracking ratios.

Average Tracking Ratio							
Scenario	Setup	BC	MTC	Optimal	Horizontal	Vertical	Greedy
	DENSE(1)	0.4761	0.9596	0.9583	-	0.9249	0.8897
	SPARSE(1)	0.3472	0.8758	0.8526	-	0.6926	0.6444
omniscient	NARROW(1)	0.5726	0.9668	0.9645	-	0.9366	0.9068
	DENSE(2)	0.4447	0.96	0.9562	0.9361	0.8689	0.8426
	SPARSE(2)	0.3181	0.8762	0.7713	0.6907	0.5126	0.4595
	DENSE(1)	0.4610	0.8814	0.8625	-	0.813	0.8105
	SPARSE(1)	0.3446	0.7174	0.6678	-	0.5674	0.5653
only-camera	NARROW(1)	0.5726	0.9205	0.9023	-	0.8546	0.8597
	DENSE(2)	0.4447	0.882	0.8385	0.8159	0.7414	0.7429
	SPARSE(2)	0.3225	0.7180	0.5378	0.5454	0.4452	0.4307

**Table A2:** Relative tracking ratio values for the omniscient and only-camera scenarios, calculated as Equation (10), averaged on all five setups. (Values of the horizontal clustering with 1-barrier were considered to be the same as those of the optimal method.)

Detection Mode	BC	MTC	Optimal	Horizontal	Vertical	Greedy
omniscient	0.0%	100.0%	95.05%	87.18%	73.54%	65.82%
only-camera	0.0%	100.0%	84.29%	78.67%	64.69%	64.07%
